# Mini Review/Commentary: Growth Hormone Treatment in Children with Type 1 Diabetes

**DOI:** 10.3390/ijms20030772

**Published:** 2019-02-12

**Authors:** Walter Bonfig, Reinhard W. Holl

**Affiliations:** 1 Department of Pediatrics, Technical University München, D-80804 Munich, Germany; 2 Department of Pediatrics, Klinikum Wels-Grieskirchen, Grieskirchner Str. 42, A-4600 Wels, Austria; 3 Institute of Epidemiology and Medical Biometry, University of Ulm, D-89019 Ulm, Germany; r.holl@uni-ulm.de; 4 German Center for Diabetes Research (DZD), D-85764 Munich-Neuherberg, Germany

**Keywords:** growth hormone, type 1 diabetes, growth hormone treatment, GH–IGF-I axis

## Abstract

In the state of insulin deficiency, the growth hormone—insulin-like growth factor-I (GH–IGF-I) axis is altered due to hepatic GH resistance, which leads to GH hypersecretion and low circulating IGF-I concentration. On the other hand, both growth hormone deficiency (GHD) and GH excess have significant influence on carbohydrate metabolism. These complex interactions are challenging in diagnosing GHD in subjects with type 1 diabetes mellitus (T1DM) and in treating subjects with T1DM with GH. So far, there is only limited clinical experience in GH treatment in patients with T1DM, but recently first reports on metabolic safety and efficacy of GH treatment in subjects with T1DM have been published.

## Mini Review/Commentary

The combination of the two diagnoses “growth hormone deficiency” (GHD) and “type 1 diabetes mellitus” (T1DM) is quite rare, given a prevalence of GHD ranging between 1:3500 and 1:8700 and an incidence of T1DM <15 years of age of 1:5000 in Germany [1,2]. Even if a child with T1DM shows a GHD suggestive growth pattern, physicians are frequently hesitant to perform regular work-up and to start growth hormone treatment in a diabetic child [3]. Uncertainty relating to diagnostic criteria (insulin-like growth factor-I (IGF-I) normal values and growth hormone cut-off in a diabetic child) and fear of worsening metabolic diabetes control might be obstacles in treating diabetic children with growth hormone. In an Italian survey of 42 pediatric endocrine centers, the majority of physicians usually avoided combined growth hormone and insulin therapy [3]. In only 13 centers in total 17 patients were treated with growth hormone under various indications (GHD, short stature without catch-up growth in children born small for gestational age, and Turner Syndrome); in nine patients, GH treatment had already been started before diabetes onset, and in eight patients, after diagnosis of T1DM [3].

GH plays an important role in glucose, lipid, and protein metabolism [4]. Both GHD and GH excess are associated with disturbances of carbohydrate metabolism. GH decreases glucose oxidation and glucose uptake by muscle and increases gluconeogenesis resulting in “insulin antagonist effects” [5]. The growth promoting effects of GH are mediated through the insulin-like growth factors (mainly IGF-I), which are synthesized and secreted by the liver as well as in target tissues. Insulin-like growth factors are bound to insulin-like growth factor binding proteins, with IGFBP-3 being the major insulin-like growth factor binding protein. IGF-I, IGFBP-3, and the acid labile subunit (ALS) form a ternary complex extending the half-life of IGF-I [5]. Adequate insulin secretion and portal insulin concentrations are needed to support normal serum concentrations of IGFs and IGF-BPs, since insulin modulates hepatic GH receptor expression. Portal insulin deficiency leads to GH hypersecretion. Despite GH hypersecretion circulating concentrations of IGF-I and IGFBP-3 are low, and concentrations of IGFBP-1—a major negative regulator of IGF-I bioactivity—is high in the state of insulin deficiency [5].

So far, very few data regarding GH treatment in GH deficient children with T1DM have been published [3,6,7]. In one study, 37 patients with GHD after the onset of T1DM in the German-Austrian DPV (“Diabetes Patienten Verlaufsdokumentation”) database were identified [6]. Longitudinal data was available for 21 patients. Over a two-year GH treatment period, mean daily insulin requirements increased significantly from 0.74 IU/kg/day to 1.1 IU/kg/day (*p* < 0.05) and was also significantly higher than in a large control group (*n* = 48,856: mean daily insulin requirement 0.85 IU/kg/day). Mean hemoglobin A1c increased from 7.6% to 8.2%, but did not differ from the large control group of children with T1DM only (*n* = 48,856: hemoglobin A1c 8.2%, *p* > 0.05). Surprisingly, over the two-year study period, a height gain of only 0.3 standard deviation scores (SDS) was observed in children with GHD and T1DM indicating decreased efficacy of GH treatment in children with T1DM, but no data on GH dosage and IGF-I concentrations was available in the database [6]. Therefore, reasons for decreased efficacy of GH treatment in children with T1DM might have been a delayed diagnosis of GHD, inadequate GH dosage, or the hepatic GH resistance in subjects with T1DM. For this reason, the same authors subsequently performed an analysis of first year growth response (catch-up growth) in children with T1DM and GHD in KIGS (Pfizer International Growth Database) [7]. In KIGS, 24 prepubescent children with pre-existing T1DM and diagnosis of GHD were identified and compared to 15,024 prepubescent controls with GHD only. First year growth response was comparable between patients with T1DM/GHD and controls (7.54 ± 3.11 cm/year versus 8.35 ± 2.54 cm/year, *p* = 0.38), and height SDS improved from −2.62 SDS to −1.88 SDS over one year of GH treatment [7]. At start of GH treatment, GH dosage was comparable (0.23 ± 0.08 mg/kg/week versus 0.22 ± 0.07 mg/kg/week, *p* = 0.09), but after one year, GH dosage was higher in subjects with T1DM and GHD (0.24 ± 0.08 mg/kg/week versus 0.21 ± 0.07 mg/kg/week, *p* = 0.04). The conclusions from these two studies were that:

(1) GH treatment is metabolically safe in diabetic subjects, if higher insulin demands are considered and insulin doses are adjusted accordingly.
(2) GH treatment is effective in diabetic children under adequate GH dosage.

Therefore, pre-existing T1DM should not be a reason to deprive children with T1DM and GHD from GH treatment. Children with well-controlled T1DM and GHD suggesting growth pattern should undergo regular “short stature work-up” even though diagnosis of GHD might be challenging in children with T1DM because of the disturbed GH–IGF-I axis with hepatic GH resistance, GH hypersecretion, and low circulating IGF-I [8,9,10]. Recently, reference values for IGF-I concentrations of children and adolescents with T1DM have been published [8], confirming lower mean IGF-I concentrations in diabetic subjects. Both poor metabolic control and diabetes duration had negative effects on serum IGF-I concentration in this study.
